# Fostering Patient Choice Awareness and Presenting Treatment Options Neutrally: A Randomized Trial to Assess the Effect on Perceived Room for Involvement in Decision Making

**DOI:** 10.1177/0272989X211056334

**Published:** 2021-11-02

**Authors:** Arwen H. Pieterse, Kim Brandes, Jessica de Graaf, Joyce E. de Boer, Nanon H. M. Labrie, Anouk Knops, Cornelia F. Allaart, Johanna E. A. Portielje, Willem Jan W. Bos, Anne M. Stiggelbout

**Affiliations:** Department of Biomedical Data Sciences, Leiden University Medical Center, Leiden, Zuid-Holland, The Netherlands; Department of Biomedical Data Sciences, Leiden University Medical Center, Leiden, NSW, The Netherlands; Department of Biomedical Data Sciences, Leiden University Medical Center, Leiden, NSW, The Netherlands; Department of Biomedical Data Sciences, Leiden University Medical Center, Leiden, NSW, The Netherlands; Athena Institute, Vrije Universiteit Amsterdam, Amsterdam, The Netherlands; Dutch Federation of Patients’ Organizations, Quality of Care Department, BM, Utrecht, The Netherlands; Department of Rheumatology, Leiden University Medical Center, Leiden, The Netherlands; Department of Medical Oncology, Leiden University Medical Center, Leiden, The Netherlands; Department of Internal Medicine, Leiden University Medical Center, Leiden, The Netherlands; Department of Internal Medicine, St. Antonius Hospital, Nieuwegein, The Netherlands; Department of Biomedical Data Sciences, Leiden University Medical Center, Leiden, NSW, The Netherlands

**Keywords:** communication, experiment, implicit persuasion, options, patient involvement, shared decision making

## Abstract

**Purpose:**

Shared decision making calls for clinician communication strategies that aim to foster choice awareness and to present treatment options neutrally, such as by not showing a preference. Evidence for the effectiveness of these communication strategies to enhance patient involvement in treatment decision making is lacking. We tested the effects of 2 strategies in an online randomized video-vignettes experiment.

**Methods:**

We developed disease-specific video vignettes for rheumatic disease, cancer, and kidney disease showcasing a physician presenting 2 treatment options. We tested the strategies in a 2 (choice awareness communication present/absent) by 2 (physician preference communication present/absent) randomized between-subjects design. We asked patients and disease-naïve participants to view 1 video vignette while imagining being the patient and to report perceived room for involvement (primary outcome), understanding of treatment information, treatment preference, satisfaction with the consultation, and trust in the physician (secondary outcomes). Differences across experimental conditions were assessed using 2-way analyses of variance.

**Results:**

A total of 324 patients and 360 disease-naïve respondents participated (mean age, 52 ± 14.7 y, 54% female, 56% lower educated, mean health literacy, 12 ± 2.1 on a 3–15 scale). The results showed that choice awareness communication had a positive (M_present_ = 5.2 v. M_absent_ = 5.0, *P* = 0.042, *η*^2^_partial_ = 0.006) and physician preference communication had no (M_present_ = 5.0 v. M_absent_ = 5.1, *P* = 0.144, *η*^2^_partial_ = 0.003) significant effect on perceived room for involvement in decision making. Physician preference communication steered patients toward preferring that treatment option (M_present_ = 4.7 v. M_absent_ = 5.3, *P* = 0.006, *η*^2^_partial_ = 0.011). The strategies had no significant effect on understanding, satisfaction, or trust.

**Conclusions:**

This is the first experimental evidence for a small effect of fostering choice awareness and no effect of physician preference on perceived room to participate in decision making. Physician preference steered patients toward preferring that option.

The importance of shared decision making has gained much traction since Charles and colleagues published their hallmark papers in 1997 and 1999.^1,2^ Much research has been published on opinions regarding shared decision making,^3,4^ its occurrence,^5,6^ barriers and facilitators,^7,8^ clinician training,^9^ and patient decision aids.^10^ No consensus exists on the precise definition of the concept, although models share common elements.^11^ Two elements seem of particular relevance for patients to become truly involved in preference-sensitive decisions: fostering choice awareness^1,12–21^ and avoidance of implicit persuasion.^1,12,13,22^ The effects of these communication strategies have not been assessed to date.

The first element is patients’ awareness that there is choice and that their views are relevant to that choice. This “choice awareness” may need to be created by explicitly acknowledging that there is more than one reasonable way to address the patient’s situation, since patients often expect clinicians to know the best option.^23^ In practice, this step is often omitted.^24^ The second element is for clinicians to avoid steering patients (implicitly) toward a particular option.^1,13,25^ This may happen when clinicians have a personally favored alternative, for example based on experience. Ideally, clinicians refrain from suggesting a favored option before patients have considered the options in light of their own preferences. Steering, or implicit persuasion, occurs frequently, even in situations of equipoise.^26–28^

We set out to test the effects of the 2 communication strategies on patients’ perceived room for involvement in treatment decision making, understanding of options, treatment preference, satisfaction with the consultation, and trust in the physician. We hypothesized that when a clinician fosters choice awareness (versus not), patients would 1) perceive more room for involvement in decision making (hypothesis 1) and 2) understand the information better (hypothesis 2), as it would encourage them to listen more actively, which has been shown to stimulate cognitive processing of information.^29,30^ When a clinician shows a treatment preference (versus not), we hypothesized that patients would 3) perceive less room for involvement in decision making (hypothesis 3) and 4) prefer that option more often, without them being aware of this influence (hypothesis 4). We had no a priori hypotheses about effects on satisfaction or trust. There is some evidence that patients’ perception of shared decision making is associated with higher satisfaction^31^ but also that raising uncertainty about the best option may decrease satisfaction^32^ and that patients like to receive advice from their clinician.^12,33^ Further, mixed results have been reported on the association between shared decision making and trust.^34–37^

We tested our hypotheses in a randomized online experiment using video vignettes (i.e., videos of a scripted medical consultation). Manipulating clinicians’ communication in real consultations can be difficult to achieve and ethically undesirable. Video vignettes offer the possibility to manipulate communication in realistic and controlled manners.^38–40^ The video vignettes show part of an enacted consultation. All communication, medical content, and appearance of the environment and actors are standardized across all versions of the video vignettes, and only the communication elements of interest are varied.^38^ Such a design disentangles the specific effects of the communication elements on the outcomes of interest. Participants are asked to view the video vignette and are instructed to imagine that they are the patient in the video (analogue patients). Evidence has shown that analogue patients can be validly used as proxies to actual patients to evaluate clinicians’ communication behavior.^41,42^

## Methods

### Study Design

An online randomized experiment was conducted in which participants (patients or members of the general population, the latter referred to as disease-naïve participants) viewed a video vignette embedded in an online survey. We manipulated choice awareness communication and preference communication in a 2 (presence/absence of physician statement on availability of choice) by 2 (presence/absence of physician statement on preferred treatment) between-subjects design (i.e., 4 experimental conditions). For sake of generalizability, we recorded the video vignettes in 3 different disease contexts: rheumatic disease, cancer (i.e., multiple myeloma or Kahler’s disease), and kidney disease (Table 1). Disease-naïve participants were randomly assigned to view 1 of the 3 disease vignettes. Patients viewed a video vignette relating to their own diagnosis. The video vignettes lasted between 3:32 and 4:11 min (rheumatic disease), 4:05 and 4:38 min (cancer), and 4:32 and 5:08 min (kidney disease) across experimental conditions. Participants could not proceed with the survey unless they had watched at least 3:20 min of the video.

**Table 1:** Number of Participants per Experimental Condition, by Disease Context Featured in the Video Vignette (*N* = 684)^a^

		Choice Awareness Communication			
		Absent		Present	
		*n* = 167		*n* = 175	
Preference communication	Absent	Rheumatic disease (%)	50 (30)	Rheumatic disease (%)	56 (32)
		Cancer (%)	68 (41)	Cancer (%)	67 (38)
		Kidney disease (%)	49 (29)	Kidney disease (%)	52 (30)
		***n* = 172**		***n* = 170**	
	Present	Rheumatic disease (%)	53 (31)	Rheumatic disease (%)	54 (32)
		Cancer (%)	67 (39)	Cancer (%)	63 (37)
		Kidney disease (%)	52 (30)	Kidney disease (%)	53 (31)

a Patient participants were assigned to the video vignette featuring their own disease and were randomized over the experimental conditions; disease-naïve participants were randomized over disease contexts featured in the video-vignette and over experimental conditions.

### Participants and Procedure

Participants were patients diagnosed with a rheumatic disease (any type), cancer (any type), or kidney disease (any stage), or members of the general population (disease-naïve participants). We expected disease-naïve participants to be able to imagine themselves being the patient,^41^ and the communication strategies to have similar effects in disease-naïve and patient participants. Participants were eligible if they were 18 years or older. Patients were recruited via advertisements of the Dutch association for patients with a rheumatic disease (Reumazorg Nederland) and via two Dutch online patient panels (kanker.nl for cancer patients, nponline.nl for kidney disease patients). Patients were eligible irrespective of: disease activity at the time of participation; time since diagnosis; and past, present or intended treatment. They received no remuneration for their participation. Disease-naïve participants were recruited via a panel agency (Kantar, formerly known as Lightspeed Research); they received vouchers worth €0.10, conforming the average length it took participants to complete the survey. The ethical committee of the Leiden University Medical Center approved the study (protocol No. P18.097).

### Video Vignettes

The scripts for the video vignettes were developed in our project team consisting of 2 rheumatologists, 2 hematologists, 2 nephrologists, and patients with rheumatic disease (*n* = 5), multiple myeloma (*n* = 2), non-Hodgkin lymphoma (*n* = 1), or kidney disease (*n* = 5). One author (K.B.) further observed consultations in all relevant specialties. The treatment options presented to the patient in the video vignette were rituximab via an infusion at the hospital every 6 mo versus etanercept in weekly injections at home (rheumatic disease); melphalan, prednisone, and bortezomib (oral medication) at home combined with a weekly injection at the hospital versus lenalidomide and dexamethasone (oral medication) at home (cancer); or hemodialysis at the hospital 3 times a week versus peritoneal dialysis at home every night (kidney disease). The video vignettes ended after the physician had described the options and before the patient’s preferences were addressed, to facilitate participants’ projection into the patient depicted.

Physicians and patients on the team were asked to provide feedback on the realism of the scripts. The authors adjusted the scripts accordingly. Then other physicians and patients were asked for feedback on the revised versions, and we adjusted the scripts again accordingly. The final versions of rheumatic disease scripts were pretested to check whether the manipulations were perceived as intended, first among 24 fourth-year medical students and then among 21 members of the general population (see Supplementary Appendix A). Based on both tests, we concluded that the manipulations were perceived as intended. The manipulations are shown in Box 1. Note that the physician explicitly stated that the 2 treatment options were equally effective in all 4 videos in the rheumatic and kidney disease contexts and unintendedly omitted this phrase in the oncology context.

**Box 1:** Choice awareness and preference communication manipulations

Choice awareness communication
“You are eligible for two types of [treatment]. They both have their own pros and cons. **Therefore it is important that we discuss what is important to you and what feels best for you. Then we can decide which [type of treatment] would suit you best.** (…) **It is therefore important to see together which of the two fits you best.**”
Preference communication
“**I have very good experiences with [treatment option X]. Patients such as you whom I see do very well on [treatment option X].** (…) **They are both good options. It is a bit less of a hassle at home if you get the drip at the hospital/It can be nicer to get possibly into remission after 12 months/It is a bit less of a hassle at home if you do the dialysis at the hospital^a^. And patients generally do very well on [treatment X].** Do you have questions at this moment?”

**In bold font:** added text in the communication strategy present conditions. The physician explicitly stated in all 4 conditions in the rheumatic and kidney disease contexts that the 2 treatment options were equally effective and unintendedly omitted this phrase in the oncology context

a The physician mentioned an advantage specific to treatment X depending on the disease context.

For the role of the physician in all videos, we hired a male actor who was experienced with acting for video-vignette studies. The patient in the rheumatic disease and cancer video vignettes was the same female actor, and the patient in the kidney disease video vignettes was a male actor; both represented a patient in her or his early 70s. A further description of the video vignettes is shown in Supplementary Appendix A.

### Measures

The participants were asked to complete background questions before viewing the video vignette and outcome and manipulation check measures afterward.

#### Background characteristics

Participants were asked to indicate their gender, year of birth, education, and their level of health literacy using three 5-point screening questions^43^ (Cronbach’s α = 0.59; range summed scores, 3–15). Patients were asked to specify month and year of diagnosis, disease type/stage, and past/current treatments. Disease-naïve participants were asked whether they had been treated for a disease that had required 4 or more hospital visits or bimonthly visits to their general practitioner in the past 5 years and, if so, what disease (i.e., diabetes, cardiovascular disease, disease of the lungs/airways, skin disease, or other to specify). Those indicating they had a rheumatic disease, cancer, or kidney disease were treated as patient participants.

#### Primary outcome measure

Perceived room for involvement in treatment decision making was measured with 4 self-developed 7-point response items (ranging from 1 = *totally not* to 7 = *very much*), adapted from an earlier study.^44^ The items asked to what extent participants expected the physician to give them space during the consultation to 1) think and 2) give their opinion about the pros and cons of the treatment options, 3) think along about the decision, and 4) that the physician considered their opinion to be important in making a treatment choice. Internal reliability was good (Cronbach’s α = 0.94; range averaged score, 1–7).

#### Secondary outcome measures

Understanding of the information about the treatment options was measured by 1) *free recall* of pros and cons of the 2 options^45^ and 2) *aspects* that participants had considered in determining their preferred option, reflecting the extent to which they understood how options differed. Free recall was assessed using 4 open-ended questions asking what the physician had told, and aspects using a preset list and an open question (Box 2). Detailed a priori scoring rules were developed per disease context to score the correctness of free recall (Supplementary Appendix B). Each unique aspect that a participant identified that was in line with the information in the video and on which the treatment options differed was considered correct. Two independent coders (A.H.P., J.E.d.B.) coded the free recall and added aspects, and determined all final scores in consensus.

**Box 2:** Aspects considered in determining participants’ treatment preference

The participants were asked to identify aspects underlying their treatment preference by selecting one or more aspects from a pre-set list and/or adding aspects relevant to them. The aspects did not point to either of the two treatment options. To illustrate, the pre-set list of aspects in the cancer context were:
□ Only pills or pills combined with an injection at the hospital
□ Hormonal therapy yes or no
□ How long the disease remains inactive
□ A risk of getting neuropathy or not
□ 12 or 16 months of treatment
Participants could select as many aspects from the list as they wished. The first, fourth and fifth aspects listed in each context distinguished the options and were in line with the information from the video, and were therefore considered to be correct if selected; the second and third did not distinguish the options and/or were not in line with the information in the video, and were therefore considered to be incorrect if selected.
The aspects added were considered to be correct if they distinguished the options, were in line with the information in the video, and were concordant with the participant’s preference. Each unique and correct aspect received one point. No points were allocated to aspects that were based on information that had not been conveyed in the video.

The participants were asked to indicate their treatment preference, imagining that they were the patient in the video, using a leaning scale^45^ ranging from 0 = *prefer [context-specific option X]* (e.g., rituximab) to 10 = *prefer [context-specific option Y]* (e.g., etanercept). Option X was the physician’s preferred option in the preference communication present conditions across disease contexts. Further, participants were asked, “To what extent would the physician’s treatment preference influence your choice?” (scores ranged from 1 = *totally not* to 7 = *very much*) and “Do you find this pleasant?” (scores ranged from 1 = *totally not* to 7 = *very much*).

Participants’ satisfaction with the consultation was measured using 1 item derived from the Patient Satisfaction Questionnaire^46^ asking, “How satisfied are you, on the whole, with this conversation?” (scores ranged from 1 = *not at all* to 5 = *very much*). Participants’ trust in the physician was measured with 1 item asking agreement with the statement, “You fully trust the physician in the video” (scores ranged from 1 = *totally disagree* to 5 = *totally agree*) and derived from the Trust in Physician Scale.^47^

#### Video engagement and manipulation checks

At the end of the survey, we measured whether participants had felt engaged in the video and could relate to the patient in the video, using the Video Engagement Scale (15 seven-point response items ranging from 1 = *totally disagree* to 7 = *totally agree*).^48^ Internal reliability was good (Cronbach’s α = 0.93; range of average scores, 1–7). As manipulation check questions, we asked the participants to indicate their agreement with the following 2 statements on a 7-point scale (1 = *totally disagree* to 7 = *totally agree*): 1) the physician explains that the patient’s opinion is important in this decision, and 2) the physician indicates which treatment option seems best.

### Sample Size

The study was powered on the primary outcome measure. In a previous (unpublished) study of our group,^49^ we found a mean score on this 7-point scale of 5.06 with a standard deviation of 1.70. To be able to detect a difference of 0.5 in an analysis of variance (ANOVA) between-subjects analysis with a power of 80% and an alpha of 5%, 91 participants per experimental condition were needed (i.e., *N* = 364 participants in total). We doubled the target number of participants to be able to investigate possible different effects of the manipulations by education level and disease context. We aimed to recruit *n* = 120 patients per disease type (i.e., *n* = 360 in total) and *n* = 360 disease-naïve participants.

### Statistical Analyses

Time spent on the survey was computed as time in minutes between starting and completing the survey and could include breaks. Free recall and aspect scores were scaled into separate percentages by relating participants’ scores to, respectively, the maximum possible recall score per context or the total number of aspects that the participant had identified. An understanding score was computed as the average of these 2 percentages (range of possible scores, 0–100).

Descriptive analyses (frequencies and means or medians as appropriate) were used to present the participants’ characteristics and scores on the manipulation check questions. A 2-way ANOVA was conducted to compare video engagement between patients and disease-naïve participants and across disease contexts.

A multiway analysis of covariance (ANCOVA) was conducted to assess the effect of choice awareness and preference communication on perceived room for involvement (hypotheses 1 and 3) and on understanding (hypothesis 2). Education was included as covariate. In these and the following models, we included as moderators disease context and whether or not a participant was disease naïve. We have included the interactions between the 2 manipulations and between the 2 manipulations and the moderators in the initial model of all analyses and removed any if they turned out to be nonsignificant. We excluded participants who scored 0 on understanding in the analysis regarding hypothesis 2, considering this score as invalid. The effect of preference communication on participants’ treatment preference (hypothesis 4) was assessed using ANCOVA, controlling for choice awareness communication and the extent to which participants had reported that a physician’s preference would influence their treatment choice. A multivariate analysis of variance (MANOVA) was conducted to assess the effect of the communication strategies on satisfaction and trust, using Pillai’s Trace to determine significance. Homogeneity of variances across ANOVA groups was tested using Levene’s test. Box’s test was used to test the similarity of the variance-covariance matrices between groups (MANOVA). If there was a significant difference between groups for an outcome variable, post hoc comparisons with a Bonferroni correction were performed. Partial eta-squared (*η*^2^_partial_) is reported as a measure of effect size.^50^ Suggested norms for interpretation are: small = 0.01, medium = 0.06, and large = 0.14.^51^ Analyses were carried out in SPSS version 26. Significance was tested at α = 0.05.

## Results

### Participants

Between October 2018 and March 2019, 360 disease-naïve participants and 324 patients took part, of whom 141 had been diagnosed with cancer, 97 with a rheumatic disease, and 86 with kidney disease. The participants completed the survey in a median of 16 min (range, 6–9780), and 93% of them in less than 1 h. Participants were 52 ± 14.7 y old on average (range, 18–87 y), 54% were female, 56% had completed less than college education, and overall mean health literacy was 12 ± 2.1. Patients had been diagnosed between 12 d and 58 y ago (median = 7 y). Table 2 shows the background characteristics by experimental condition and shows these to be equally distributed across conditions.

**Table 2:** Participants’ Sociodemographic and Disease Characteristics by Experimental Condition (*N* = 684)

		Communication About …				
		Choice Awareness NO/Preference NO (*n* = 167)	Choice Awareness YES/Preference YES (*n* = 170	Choice Awareness NO/Preference YES (*n* = 172)	Choice Awareness YES/Preference NO (*n* = 175)	Test of Group Differences^a^; *P* Value
Mean age (SD)		51.8 (14.9)	52.8 (13.7)	51.3 (15.8)	52.6 (14.6)	0.773
Female gender (%)		96 (58)	84 (49)	95 (55)	93 (53)	0.493
Lower education (%)		96 (58)	94 (55)	93 (54)	100 (57)	0.888
Health literacy (SD)		11.8 (2.07)	11.9 (2.09)	12.0 (2.07)	11.8 (2.03)	0.755
Patient (%)		82 (49)	82 (48)	78 (45)	82 (47)	0.907
Diagnosis (%)	Rheumatic disease^b^	23 (14)	25 (15)	24 (14)	25 (14)	0.999
	Cancer^b^	37 (22)	34 (20)	25 (15)	35 (20)	
	Kidney disease^b^	22 (13)	23 (14)	19 (11)	22 (13)	
	Other^c^	24 (14)	22 (13)	25 (15)	20 (11)	
	None^c^	61 (37)	66 (39)	69 (40)	73 (42)	

a Differences between conditions were tested using analysis of variance, chi-square test, or Kruskal-Wallis test, as appropriate.

b These are all patient participants. They could have any form or stage of the disease.

c These are all disease-naïve participants. They could report no disease, 1 disease, or >1 disease, using 1 of the following categories: diabetes, cardiovascular disease, lung/airways disease, skin disease, other, none. Numbers in any of the named disease categories added up to *n*≤ 5 (3%), the other category to *n*≤ 17 (10%).

### Engagement in the Videos and Manipulation Checks

Mean scores on the Video Engagement Scale ranged from 3.9 (patients, cancer context) to 4.4 (disease-naïve participants, cancer context) and did not significantly differ between patients and disease-naïve participants or between participants across the 3 disease contexts (data not shown). The manipulations in the video vignettes were perceived as intended; those who had versus had not been exposed to the respective communication strategies scored higher on the corresponding manipulation measure (choice awareness, M = 5.0 v. 4.4, *t* = −4.0, *P* < 0.001; preference, M = 4.9 v. 3.6, *t* = −11.2, *P* < 0.001).

### Overall Perceived Room for Involvement, Understanding, Preference, Satisfaction, and Trust

The mean perceived room for involvement in treatment decision making was 5.1 (SD = 1.4) overall. Median understanding was 44% (range, 0%–72%); the median free recall subscore was significantly lower than the median aspect subscore (Md_recall_ = 11%, Md_aspect_ = 67%, Sign test, *P* < 0.001). Fifty-five (8%) participants scored 0 on understanding; they were on average younger (M = 45 v. 53 y, *t* = 3.05, *P* = 0.003), higher educated (58% v. 43%, χ^2^ = 4.9, *P* = 0.033), and less health literate (M = 10.9 v. 12.0, *t* = 2.97, *P* = 0.004). They did not significantly differ in gender or disease experience. In total, 510 of 684 (75%) participants identified at least 1 correct aspect (range, 1–4). One hundred ten (16%) participants did not reveal a treatment preference, and 289 of the remaining 574 (50%) reported a preference for the treatment option that the physician had favored in the preference communication present conditions. Overall, most (604/684, 88%) reported that a physician’s treatment preference would influence their own preference at least to some extent (score ≥4 on a scale of 1–7), and 19 of 684 (3%) stated that it would not at all. Most (555/684, 81%) also indicated that they would appreciate this influence (score ≥4 on a scale of 1–7) and 26 of 684 (4%) not at all. Mean scores for influence and appreciation were 4.9 (SD = 1.3) and 4.6 (SD = 1.4), respectively. Overall, mean satisfaction was 3.5 (SD = 1.0), and mean trust was 3.7 (SD = 0.9).

### Effect of Choice Awareness and Preference Communication

Mean perceived room for involvement was significantly higher when choice awareness communication was present versus absent (hypothesis 1 confirmed; Table 3). Supplementary Appendix C shows differences in mean scores by disease context by hypothesis. A separate analysis in the oncology context (*n* = 265) also confirmed hypothesis 1 (M_present_ = 5.3 v. M_absent_ = 4.9, *F*[*df*, error] = 5.2[1, 260], *P* = 0.023, *η*^2^_partial_ = 0.020). The effect of preference communication was not significant (hypothesis 3 rejected). Choice awareness communication had no significant effect on understanding (hypothesis 2 rejected). In the oncology setting, the effect was also in the expected direction but again not significant (M_present_ = 31.5 v. M_absent_ = 28.2, *F*[*df*, error] = 1.6[1, 234], *P* = 0.21, *η*^2^_partial_ = 0.007). Preference communication significantly predicted participants’ treatment preference (hypothesis 4 confirmed). That is, significantly more participants preferred the option favored by the physician if the physician had expressed a treatment preference than if he had not (Table 3), controlling for the extent to which the participants judged that the physician’s treatment preference would influence them. Note that this judgment had no significant effect on their preference (*F*[*df*, error] = 1.3[1, 677], *P* = 0.25, *η*^2^_partial_ = 0.002). MANOVA showed no significant effect of choice awareness (Pillai’s Trace = 0.003, *F*[*df*, error] = 0.9[2, 670], *P* = 0.42, *η*^2^_partial_ = 0.003) or preference (Pillai’s Trace = 0.007, *F*[*df*, error] = 2.2[2, 670], *P* = 0.110, *η*^2^_partial_ = 0.007) communication, on satisfaction or trust.

**Table 3:** Scores (Means, Standard Errors) for Perceived Room for Involvement in Decision Making, Understanding, and Treatment Preference by Presence and Absence of Choice Awareness and Preference Communication, Test Values, and Significance (*N* = 684)^a^

	Choice Awareness Communication					Preference Communication				
	Absent (*n* = 339)	Present (*n* = 345)	*F*(*df*, Error)	*P*	η^2^_partial_	Absent (*n* = 342)	Present (*n* = 342)	*F*(*df*, Error)	*P*	η^2^_partial_
	M (SE)	M (SE)				M (SE)	M (SE)			
Perceived room for involvement in decision making (scale, 1–7)	5.0 (0.08)	5.2 (0.07)	4.1 (1, 677)	0.042	0.006	5.1 (0.07)	5.0 (0.07)	2.1 (1, 677)	0.144	0.003
Understanding of information	39.6 (0.97)	41.5 (0.96)	1.8 (1, 622)	0.183	0.003					
Treatment preference						5.3 (0.16)	4.7 (0.16)	7.6 (1, 677)	0.006	0.011

a SE = standard error; *df* = degrees of freedom; *η*^2^_partial_ = partial eta-squared. The interactions between choice awareness and preference communication and between choice awareness and preference communication and the moderators (disease context, being v. not being disease-naïve) were not significant and were therefore removed from the final models.

## Discussion

This is the first study to collect experimental evidence for the effect of 2 communication strategies expected to assist shared decision making^11^: fostering choice awareness and refraining to present a favored option to support the neutral presentation of options. Two hypotheses were confirmed: choice awareness communication appears to foster perceived room for involvement in decision making and does so regardless of education or diagnosis; and communicating clinician’s preferred option steers patients toward that option. Effect sizes were small, in line with meta-analyses that have shown effect sizes in studies on effectiveness of messages to be typically small.^52^ In addition, experimental video-vignette studies offer good evidence on directions of effects but not necessarily on their size in clinical practice.^53^ The mean difference of choice awareness communication on perceived room for involvement was larger when the physician did not state that the 2 options were equally effective (i.e., in the oncology context), suggesting that this statement already conveyed choice. The findings support the theoretical validity of including the 2 communication strategies in models of shared decision making. Choice awareness communication did not affect patient understanding, and stating the clinician’s treatment preference did not affect perceived room for involvement. Neither communication strategy affected satisfaction with the consultation or trust in the physician. Further testing of the communication strategies should determine the robustness of the findings.

The implementation of the 2 strategies, to raise patient engagement in decision making and to avoid steering patients toward an option for which they may not share a preference, requires clinicians to be convinced of the importance of the personal views of patients in deciding what is best. Clinicians may act on what they think is best for patients, rather than explore what patients actually want.^54^ Further, clinicians’ attitudes toward shared decision making tend to be largely positive but also coupled with reluctance to share decisional control and a lack of understanding of the concept.^55,56^ Forcino et al.,^55^ for example, found that among 272 US-based clinicians involved in family medicine or surgery, up to half reported feeling uncomfortable with decisions that stray away from what they think is clinically most appropriate. Also, less than half defined shared decision making in terms of patients and clinicians involved in making decisions together. Comparably, one-third of 112 Dutch trauma surgeons defined shared decision making in ways clearly discordant with current consensus, such as describing a classic process of obtaining informed consent or indicating that patients need to make the final decision on their own.^56^ The communication strategies themselves may not be difficult to understand, but identifying when they are essential or how to display them may be. We expect clinicians to need training to that effect, as the behaviors are rarely observed in clinical practice.^23,24,26–28^ What an effective clinician training should look like is highly uncertain, though.^9^ Yet, a recent trial in which trained oncologists treating palliative care patients were compared with untrained counterparts demonstrated that training can result in significantly higher levels of fostering choice awareness.^57^

Almost 9 in 10 participants indicated that the treatment preference of the physician would influence their own, and 8 in 10 indicated that they would be OK with that; remarkably, this appreciation was true also for half of those who reported that it would not influence them (42/80, 53%). This approval is in line with studies showing that cancer patients prefer to receive a recommendation from their clinician when they face a preference-sensitive treatment decision^12^ and with findings among analogue primary care patients who felt more involved in decision making and trusted their general practitioner more if he or she had provided arguments for the treatment recommendation.^58^ Possibly, this approval also explains why hearing the clinician’s preference did not lower participants’ perceived room to participate in decision making. Importantly, this study shows that a clinician’s preference implicitly steers patients toward that option: it affects the patients’ preference regardless of the influence that the participants indicated to be aware of. Providing a recommendation can thus perfectly fit a shared decision process, if it is not given too early in the process and incorporates the patient’s considerations.^59^

We did not find an association between the communication strategies and patients’ understanding, satisfaction, or trust. The clinician stated in all 4 conditions that the patient was eligible for 2 treatment options and that both had pros and cons (Box 1). The project team agreed that this would reflect natural conversations in clinical practice in preference-sensitive decision situations. This may help explain why the overall scores on perceived room for involvement were high and possibly why further fostering choice awareness did not affect understanding. The effect on understanding was in the expected direction but may have been too small to detect. Understanding was low, overall. It was conceived of as a combination of free recall of the pros and cons of the options, which was generally poor, and aspects that distinguished the options and were personally relevant in determining preference. From other studies, it is known that it is harder for participants to reproduce information actively than to recognize correct answers.^60,61^ In addition, both sets of information required a combination of recall (What did the physician tell?) and of evaluation (Do I consider it as a pro/con and is it relevant to my preference?), which may help explain why the free recall subscores in the present study were lower than recall scores in comparable experiments.^62^ Considering aspects that distinguish alternative options is a core task when choosing which option fits best. Most participants in the current study were able to identify at least 1 correct aspect. At the same time, 1 in 6 participants did not reveal a treatment preference; they did not significantly differ from other participants in terms of age, gender, education, health literacy, or disease experience. We assume that the main reason for not reporting a preference was the short time frame to consider their preference. Further, based on these results, introducing uncertainty about the best option and/or showing a clinician’s preference may be expected to be of no consequence regarding patients’ satisfaction about the consultation or their trust in the clinician. Clearly, only part of the consultation was shown in the present study. How interactions further develop in clinical practice, and in particular, the extent to which patients feel heard and supported, may affect how involved they feel in decision making, how satisfied they are about the encounter, and how much they trust the clinician.

The manipulations were perceived as intended, but the perceived differences between experimental conditions were relatively small. Differences in perceptions of choice awareness communication were smaller in the experiment than in the pilot test, whereas those for preference communication were larger. The 2 tests differed in sample (medical students in the pilot test v. patients and disease-naïve participants in the experiment) and materials (scripts on paper v. videos). The manipulation checks in the pilot test were therefore good indicators of whether the manipulations were perceived as intended but did not exactly mimic the test situation. Importantly, the videos seemed as realistic to patients as to disease-naïve participants and as realistic across disease contexts.

The strengths of the current study include the use of an experimental design, which allows for controlling all communication elements in the video other than the manipulations. Further, half of the participants were lower educated, which is in line with the 60% lower educated members of the Dutch general population^63^ and makes the results relevant to actual patient populations. Also, the results were found in a sample combining different disease contexts, which may be seen as an indication that they are generalizable. Importantly, the study included 3 diseases, which represents only a very small selection of potential diseases. Potential effects by disease context should be further studied, as could those by preferred decisional role. Some limitations should be noted, too. First, we powered the study on perceived room for involvement, assuming a 0.5 difference on a 7-point scale. The observed effects were much smaller but statistically significant. The clinical relevance of such a small difference is questionable. At the same time, effects in actual practice may be larger, as it is quite unusual for clinicians to make explicit that patients’ opinions matter in deciding on the best option. Second, the physician explicitly stated that the 2 treatment options were equally effective in all 4 videos in the rheumatic and kidney disease contexts but unintendedly omitted this phrase in the oncology context. The separate analysis in the oncology context suggests that stating equal effectiveness diminished the effect of the choice awareness manipulation in the other 2 disease contexts. Third, the internal validity of the findings is high, but the external validity needs further assessment. Analogue patients’ experiences are inherently different from real-life experiences, although engagement in the videos did not significantly differ between participants with and without personal disease experience.

## Conclusion

This is the first experimental evidence for a small effect of explicitly stating choice and the importance of patients’ views on perceived room to participate in decision making in a preference-sensitive decision situation in specialty care. We further experimentally showed how stating a clinician’s favored treatment option steers patients toward that option. This finding underlines the importance of incorporating patients’ considerations in clinicians’ recommendations. The challenge lies in implementing the communication strategies each time when patients’ personal views are essential in selecting the most appropriate treatment.

## Supplemental Material

sj-docx-1-mdm-10.1177_0272989X211056334 – Supplemental material for Fostering Patient Choice Awareness and Presenting Treatment Options Neutrally: A Randomized Trial to Assess the Effect on Perceived Room for Involvement in Decision Making Click here for additional data file. Supplemental material, sj-docx-1-mdm-10.1177_0272989X211056334 for Fostering Patient Choice Awareness and Presenting Treatment Options Neutrally: A Randomized Trial to Assess the Effect on Perceived Room for Involvement in Decision Making by Arwen H. Pieterse, Kim Brandes, Jessica de Graaf, Joyce E. de Boer, Nanon H. M. Labrie, Anouk Knops, Cornelia F. Allaart, Johanna E. A. Portielje, Willem Jan W. Bos and Anne M. Stiggelbout in Medical Decision Making

sj-docx-2-mdm-10.1177_0272989X211056334 – Supplemental material for Fostering Patient Choice Awareness and Presenting Treatment Options Neutrally: A Randomized Trial to Assess the Effect on Perceived Room for Involvement in Decision Making Click here for additional data file. Supplemental material, sj-docx-2-mdm-10.1177_0272989X211056334 for Fostering Patient Choice Awareness and Presenting Treatment Options Neutrally: A Randomized Trial to Assess the Effect on Perceived Room for Involvement in Decision Making by Arwen H. Pieterse, Kim Brandes, Jessica de Graaf, Joyce E. de Boer, Nanon H. M. Labrie, Anouk Knops, Cornelia F. Allaart, Johanna E. A. Portielje, Willem Jan W. Bos and Anne M. Stiggelbout in Medical Decision Making

sj-docx-3-mdm-10.1177_0272989X211056334 – Supplemental material for Fostering Patient Choice Awareness and Presenting Treatment Options Neutrally: A Randomized Trial to Assess the Effect on Perceived Room for Involvement in Decision Making Click here for additional data file. Supplemental material, sj-docx-3-mdm-10.1177_0272989X211056334 for Fostering Patient Choice Awareness and Presenting Treatment Options Neutrally: A Randomized Trial to Assess the Effect on Perceived Room for Involvement in Decision Making by Arwen H. Pieterse, Kim Brandes, Jessica de Graaf, Joyce E. de Boer, Nanon H. M. Labrie, Anouk Knops, Cornelia F. Allaart, Johanna E. A. Portielje, Willem Jan W. Bos and Anne M. Stiggelbout in Medical Decision Making
