# Supplementary material for: Fostering Patient Choice Awareness and Presenting Treatment Options Neutrally: A Randomized Trial to Assess the Effect on Perceived Room for Involvement in Decision Making
Source: Med Decis Making. 2021 Nov 2;42(3):375–86. doi: 10.1177/0272989X211056334 (PMC8918871; doi:10.1177/0272989X211056334)
Supplement: sj-docx-1-mdm-10.1177_0272989X211056334 – Supplemental material for Fostering Patient Choice Awareness and Presenting Treatment Options Neutrally: A Randomized Trial to Assess the Effect on Perceived Room for Involvement in Decision Making [file sj-docx-1-mdm-10.1177_0272989X211056334.docx]

**Appendix A. Pilot-test and Description of the video-vignettes**

**Pilot-test of the video-vignettes**

The final versions of two rheumatic disease scripts (Choice awareness communication *present*/Preference communication *absent* and vice versa) were pre-tested to check whether the manipulations were perceived as intended, first among 24 fourth-year medical students. We asked them to specify on a seven-point scale (1, ‘*Totally disagree*’ to 7, ‘*Totally agree*’) whether they agreed that the physician explained that a decision would be made and the patient’s opinion was important (Choice awareness manipulation) and that the physician offered an opinion on the best treatment when describing the treatment options (Preference manipulation). The results showed that the students who had *versus* had not been exposed to the respective communication strategies scored higher on the manipulation measures (Choice awareness, M*=*4.7 vs. 4.1; Preference, M=5.9 vs. 4.3). We slightly adapted the scripts, and then piloted these among 21 members of the general population. Again, the results showed that participants who had *versus* had not been exposed to the respective communication strategies scored higher on the corresponding manipulation measure (Choice awareness, M*=*4.8 vs. 2.8; Preference, M=4.2 vs. 3.7). We concluded that the manipulations were perceived as intended because in both pre-tests mean differences of 0.5 on a seven-point scale were detected.^1^

**Description of the video-vignettes**

The video started with a close-up view on the patient and physician seated at a table and talking to each other, and a voice-over providing information about the patient: his/her name, age, whether s/he was married and had (grand-)children, when s/he had been diagnosed, how the patient was doing physically, what the medical options were to treat the condition, that the reason for the present consultation was to discuss treatment options, that the video-vignette showed them halfway a conversation in which the patient had told the physician how s/he feels and that s/he would like to learn more about the next steps. The text of the voice-over was simultaneously shown on the screen, next to video of the patient and physician (see screenshot below for an illustration). The camera was directed alternately on the patient and physician during the video, depending on who was talking. Each video-vignette ended after the physician had explained the benefits and harms of the two options, had asked if everything was clear, and if the patient had questions, at which point the image and sound faded out. We chose to avoid the patient-actor to express any treatment preference, in order to make it easier for participants to put themselves in the place of the patient.

**Screenshot of the start of the rheumatic disease video-vignette (original Dutch language)**


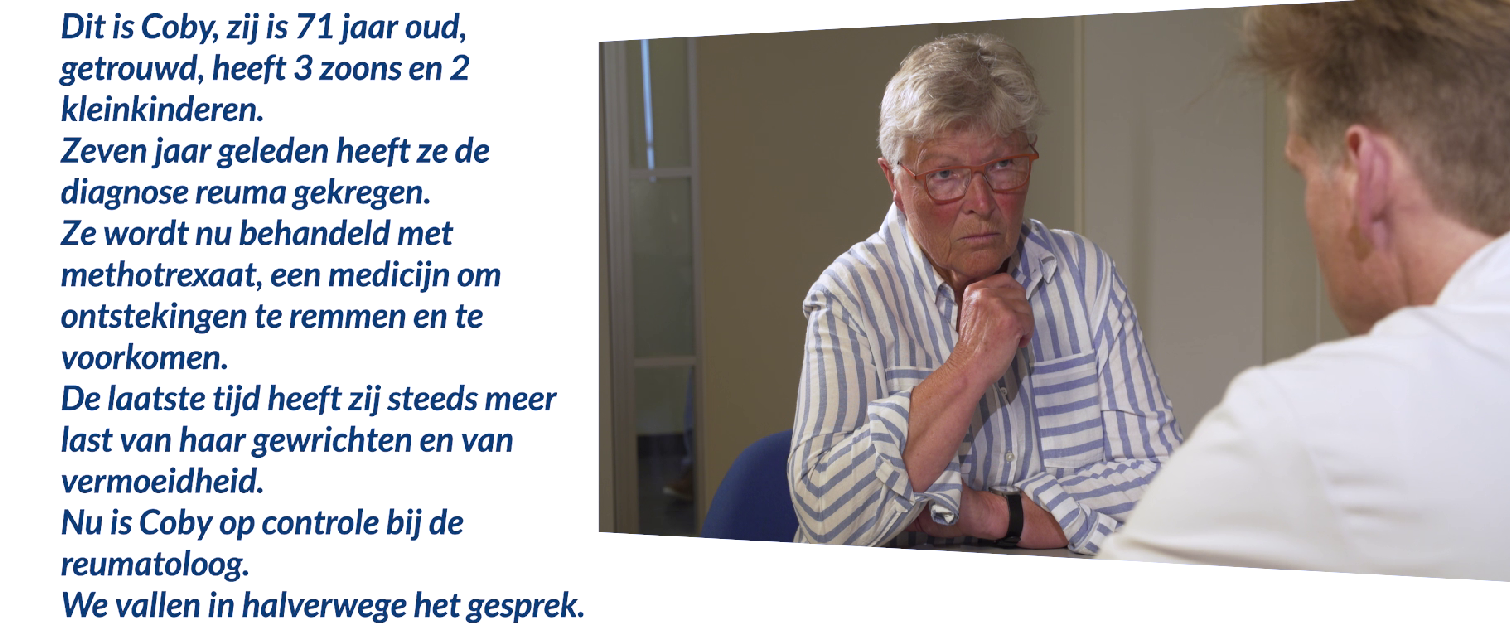


*Translation:* This is Coby, she is 71 years old, is married, has three sons and two grandchildren. She was diagnosed with rheumatic disease seven years ago. She is being treated with methotrexate, a drug that aims to slow down and prevent inflammations. She has increasingly been suffering from join pain and tiredness recently. Coby is now seeing her rheumatologist for a check-up. We listen in halfway the conversation.

In each disease setting the video-vignette in the control condition was the first to be video-recorded. The manipulations were video-recorded separately and then added to the control version, in order to keep all other elements in the video-vignette identical across conditions, except the manipulations. The actors were aware of the main aims of the study and were asked to project themselves into the person of the physician or patient, as appropriate.

**Reference**

1. Norman GR, Sridhar FG, Guyatt GH, Walter SD. Relation of distribution- and anchor-based approaches in interpretation of changes in health-related quality of life. Med Care 2001;39:1039-47.
