# Supplementary material for: Fostering Patient Choice Awareness and Presenting Treatment Options Neutrally: A Randomized Trial to Assess the Effect on Perceived Room for Involvement in Decision Making
Source: Med Decis Making. 2021 Nov 2;42(3):375–86. doi: 10.1177/0272989X211056334 (PMC8918871; doi:10.1177/0272989X211056334)
Supplement: sj-docx-2-mdm-10.1177_0272989X211056334 – Supplemental material for Fostering Patient Choice Awareness and Presenting Treatment Options Neutrally: A Randomized Trial to Assess the Effect on Perceived Room for Involvement in Decision Making [file sj-docx-2-mdm-10.1177_0272989X211056334.docx]

**Appendix B. Coding rules to score free recall**

We developed a priori context-specific coding rules to code the correctness of participants’ answers to the *free recall* questions. We based the rules on the information about the treatment options in the video and on the responses of N=110 disease-naïve participants who had participated in a pre-test of the survey, and who were not included in the final sample (N=39, rheumatic disease context; N=36, kidney disease context; N=35, cancer context).

- Participants’ responses were itemized into single information items that each received a score between 0 and 1.
- Similar information items were scored once only.
- Maximum possible correct recall scores differed between pros and cons and per disease context, depending on how much information about the options had been included in the video-vignette. Maximum scores ranged from 5.5 (benefits of Rituximab, rheumatic disease) to 11 (harms of hemodialysis, kidney disease).
- Points were allocated if the participant’s information was:

1. correct and listed under a pro or con in line with the video-vignette (1 point)
2. correct (as in 1) but described in vague terms (0.5 point)
3. correct, referred to a procedural aspect of treatment (e.g., administered at the hospital, dosage), and had not been identified as a pro or con in the video-vignette (0.5 point)
4. correct but true for both options (e.g. treatment included oral medication) (0.5 point), or
5. correct, referred to a treatment benefit (or harm) but had not explicitly been listed as such in the video-vignette, and was listed under the benefits (or harms) (0.5 points).

- No points were allocated if: the information was incorrect; was correct but referred to a treatment benefit (or harm) and was listed under the harms (or benefits); had not been conveyed in the video-vignette; or if the response was a random sequence of letters.
- Inter-rater reliability was calculated per disease context and before consensus discussion on the combined total scores over the two treatments of 20% of participants, and assessed using Krippendorff's alpha reliability estimate. The results showed high reliability (range kalpha, .95-.99), suggesting that the scoring rules were clear.
