# Supplementary material for: Fostering Patient Choice Awareness and Presenting Treatment Options Neutrally: A Randomized Trial to Assess the Effect on Perceived Room for Involvement in Decision Making
Source: Med Decis Making. 2021 Nov 2;42(3):375–86. doi: 10.1177/0272989X211056334 (PMC8918871; doi:10.1177/0272989X211056334)
Supplement: sj-docx-3-mdm-10.1177_0272989X211056334 – Supplemental material for Fostering Patient Choice Awareness and Presenting Treatment Options Neutrally: A Randomized Trial to Assess the Effect on Perceived Room for Involvement in Decision Making [file sj-docx-3-mdm-10.1177_0272989X211056334.docx]

**Appendix C.** Contrasts and significance for the moderator Disease context by hypothesis^b^ (N=684)

|  | **Hypotheses 1, 3^a^** | | **Hypothesis 2** | | **Hypothesis 4** | |
| --- | --- | --- | --- | --- | --- | --- |
| **Disease context** | Contrast (error) | *P*-value | Contrast (error) | *P*-value | Contrast (error) | *P*-value |
| rheumatic disease vs cancer | 0.19 (0.13) | .139 | -18.6 (1.80) | .000 | -0.30 (0.27) | .274 |
| rheumatic disease vs kidney disease | -0.43 (0.13) | .001 | 4.88 (1.90) | .010 | -0.39 (0.29) | .173 |
| cancer vs kidney disease | -0.24 (0.13) | .059 | -13.7 (1.82) | .000 | -0.69 (0.28) | .013 |

^a^ Hypotheses 1 and 3 were tested using one and the same model.

^b^ Hypothesis 1: When a clinician fosters choice awareness (versus not), patients perceive more room for involvement in decision making;

Hypothesis 2: When a clinician fosters choice awareness (versus not), patients understand the information better;

Hypothesis 3: When a clinician shows a treatment preference (versus not), patients perceive less room for involvement in decision making;

Hypothesis 4: When a clinician shows a treatment preference (versus not), patients prefer that option more often, without them being aware of this influence.
